# Relationships between professional mission, work engagement, spiritual climate, and job performance among Chinese clinical nurses: A moderated mediation model

**DOI:** 10.1371/journal.pone.0337329

**Published:** 2025-12-15

**Authors:** Jijun Wu, Yuxin Li, Xiaoli Zhong, Qin Lin, Guiqiong Xie, Zhenfan Liu, Xian Rong, Lin He

**Affiliations:** 1 Department of Nursing, Deyang People’s Hospital, Deyang, China; 2 Shulan International Medical College, Zhejiang Shuren University, Hangzhou, China; 3 Sichuan Nursing Vocational College, Chengdu, China; Ajman University, UNITED ARAB EMIRATES

## Abstract

**Background:**

With the global socio-economic development, health concerns are increasing, prompting healthcare professionals to provide higher quality healthcare services. Job performance is a behavior related to performance and competence and reflects nurses’ efficiency, quality, creativity, and goal accomplishment at work. A high level of job performance is essential for nurses, patients, and organizations. However, the overall level of nurses’ job performance is low due to several factors. This study aimed to investigate the role of clinical nurses’ sense of professional mission as a direct influence on job performance, as well as the mediating role of work engagement and the moderating role of spiritual climate.

**Methods:**

From February 2024 to March 2024, 15 tertiary hospitals and ten secondary hospitals in Sichuan Province were selected as survey respondents for clinical nurses using convenience sampling. The Professional Mission Scale, Work Engagement Scale, Spiritual Climate Scale, and Job Performance Scale were used for data collection. Model 4 and Model 7 in the SPSS 26.0 macro program were used for mediation and mediation effect analysis with moderation.

**Results:**

The results showed that sense of professional mission, work engagement, and spiritual climate were significantly and positively correlated with job performance (*P* < 0.01). Work engagement partially mediates the relationship between professional mission and work performance. Specifically, professional mission can directly influence work performance or indirectly affect it through the mediating role of work engagement. The mediating effect accounts for 29.6% of the total effect. Meanwhile, spiritual climate buffered the positive impact of a sense of professional mission on work engagement and played a moderating role in the first half path of the mediation effect model of a sense of professional mission affecting work performance through work engagement.

**Conclusion:**

This study further understood the current status of clinical nurses’ job performance in China and confirmed that a sense of professional mission positively predicted job performance. Work engagement and spiritual climate further explained the relationship between a sense of professional mission and job performance through mediating and moderating effects. Nursing managers can improve the impact of a sense of professional mission on job performance by increasing spiritual climate and clinical nurses’ work engagement.

## Introduction

It is well known that nurses, as the largest occupational group in global health care, play a vital role in maintaining and promoting people’s health. In recent years, along with the accelerated global aging process and people’s continuous emphasis on the quality of health, the demand for nurses has increased significantly. However, due to the particular nature of the nursing job, which has led to a severe turnover of nurses, the shortage of nurses has become a global problem [1]. A 2023 report by the International Council of Nurses predicts that the global shortage of nurses will reach 13 million by 2030 [2]. As the world’s most populous developing country, China faces a particularly severe nursing shortage. By the end of 2022, China’s registered nurses per 1,000 population was below the global level of 3.69 nurses per 1,000 in 2018 [3]. In addition, as nursing work is characterized by high intensity, high load, high pressure, and high risk, it is easy to cause nurses to suffer from empathy fatigue and burnout, increasing their willingness to leave their jobs and further aggravating the shortage of nursing human resources [4]. The shortage of nursing human resources not only brings significant challenges to the quality of nursing services and patient safety but also increases nurses’ workload and work-related fatigue, thus affecting the efficiency and quality of nurses’ work and leading to lower job performance [5]. At the same time, the COVID-19 global pandemic has also put nurses’ work environment and physical and mental health at significant risk, further decreasing nurses’ sense of job security and leading to low levels of job performance [6]. Job performance has always been regarded as an essential factor in measuring employee and organizational effectiveness, reflecting employees’ efficiency, quality, creativity, and goal achievement at work [7]. A high level of job performance not only helps to improve the quality of medical services and nurses’ job satisfaction but also has a positive significance in reducing burnout and nursing turnover [8]. Therefore, understanding the current situation of clinical nurses’ work performance and analyzing the relevant factors and mechanisms affecting work performance are not only the focus of nursing managers but are also of great significance for further improving the quality of nursing services and ensuring patient safety.

As a multidimensional concept, job performance focuses on employee behaviors and outcomes related to achieving organizational goals. Nurses’ job performance mainly consists of task performance and contextual performance [7]. Task performance refers to the nurse’s efforts to complete the job and obtain results closely related to the content of the job. Contextual performance refers to the behavior that promotes nurses to achieve organizational goals and is closely related to the organizational characteristics of work performance, such as creating an excellent organizational climate, maintaining a positive mindset, and good interpersonal relationships. The level of nurses’ job performance directly affects the quality and efficiency of their work and influences their career success [9]. In addition, job performance is also related to organizational trust among colleagues, psychological security, work behaviors, and role stress, which indirectly affects job well-being, innovative behaviors, and organizational commitment [10]. On the other hand, low levels of job performance also tend to trigger unsafe workplace behaviors among nurses and increase adverse patient events [11]. Thus, job performance is of great significance to the organization, nurses, and patients, affecting the growth and development of nurses’ careers and directly influencing the quality of healthcare services and patient satisfaction.

Scholars at home and abroad have conducted a series of explorations on the factors that influence nurses’ job performance. Still, they mainly focus on leadership style, professional identity, work environment, and doctor-patient relationship [11,12]. In contrast, relatively few studies have been conducted on the mechanism of the synergistic effect between nurses’ intrinsic motivation and extrinsic environment on work performance. Self-determination theory suggests that employees’ inherent motivation determines work attitudes and behaviors [13]. As a personal psychological resource, the sense of professional mission is a positive intrinsic motivation, which reflects employees’ intense passion and power for the work they are engaged in and can help individuals effectively cope with work pressure and traumatic stress to maintain work motivation and creativity [14]. Work engagement, as a positive work state, reflects an individual’s passion and commitment to their work and the energy and effort they put into their work. It is a positive quality necessary for achieving career success [15]. A positive, healthy, and supportive work environment can reduce employees’ work pressure and improve their work efficiency and creativity. Spiritual climate refers to the spiritual and cultural atmosphere of an enterprise that encourages employees to express their feelings in the workplace, which is conducive to cooperation and communication between individuals and team members, as well as the establishment of a relationship of trust, thereby alleviating work pressure and burnout, and enhancing work vitality and dedication, thereby increasing work engagement [16]. Previous studies have confirmed that nurses’ perceptions of work orientation and sense of value affect their work status in the work domain and that viewing work as the purpose and meaning of one’s life leads to high work performance [17]. However, the level of job performance is subject to change, i.e., the level of job performance is inextricably linked to the work atmosphere and motivation of the individual.

To summarize, a sense of professional mission is essential to an individual’s work motivation. It drives the power of an individual’s action and enhances work commitment, which may impact job performance. Nurses’ teamwork ability and work efficiency in an excellent spiritual climate are high, which can better stimulate nurses’ enthusiasm and motivation to work and enhance work engagement. Based on this, we examined the relationship between professional mission and nurses’ work performance from the perspective of positive psychology, taking professional mission as an antecedent variable of nurses’ job performance. Meanwhile, given that the positive impact of the professional mission on work engagement has been confirmed and that a good spiritual and cultural climate helps to enhance nurses’ work vigor and dedication, we attempted to further explore the mediating and moderating roles of work engagement and spiritual climate in the relationship between nurses’ sense of professional mission and job performance, to provide a theoretical basis for nursing administrators to develop intervention strategies to improve nurses’ job performance.

## Background

### Sense of professional mission and clinical nurse job performance

A professional mission refers to an individual’s conviction that their occupation can make life meaningful and that they are passionate and relentless in their work to realize self-worth and serve society [14]. Its connotations include orienting force, charitable contribution, significance, and value. Individuals with a strong sense of mission will make their personal goals closely linked to organizational goals, have more positive intrinsic motivation, and produce many positive psychological states, such as higher job happiness, organizational commitment, organizational cultural identity, and constructive behaviors, thus showing more positive work behaviors and outcomes. In addition, professional mission, as an essential component of positive psychology, can also help individuals maintain positive psychological states and reduce stress levels during stressful events, thereby reducing burnout [18]. Self-determination theory, as one of the essential theories in organizational behavior, emphasizes that individuals’ attitudes and behaviors are influenced by intrinsic motivation and considers intrinsic drive as the source of motivation for self-determined behaviors and the basis for generating positive behaviors and outcomes [13]. Self-determination theory highlights three innate psychological needs: autonomy (the sense that one’s actions are self-directed), competence (the sense of being able to cope with challenges), and relatedness (the sense of connection and acceptance by others). When these needs are met, individuals experience high-quality intrinsic motivation [13]. A sense of professional mission reflects an individual’s perception of their work as purposeful and meaningful. It serves as a significant intrinsic motivator, positively influencing career perceptions and work engagement [14]. Nurses with a strong professional mission base their work behaviors on a deep recognition of nursing’s inherent value (autonomy). They believe in their professional competence to save lives and heal the sick (competence). They also establish emotional connections with patients, colleagues, and the organization (satisfying the need for belonging). Therefore, according to self-determination theory, professional mission becomes a powerful, self-determined intrinsic motivator. This resource energizes individuals and fosters dedication, leading to higher job performance. Zhou et al.’s study showed that healthcare workers’ professional mission was positively correlated with their job performance and could predict job performance positively [19]. Therefore, to better understand nurses’ sense of professional mission and job performance, we propose the following hypotheses:

H1: Clinical nurses’ sense of professional mission is positively related to job performance.

### Mediating role of work engagement

As a central concept in positive psychology, work engagement has garnered increasing attention from managers and researchers alike. It refers to a positive, fulfilling, and work-related state of mind characterized by vigor, dedication, and absorption [15]. Individuals with high work engagement fully invest their physical, cognitive, and emotional resources into their work roles. Elevated work engagement is associated not only with higher job satisfaction and career fulfillment but also with improved nursing quality and patient safety. The Job Demands-Resources (JD-R) model, a widely recognized framework in occupational health psychology, classifies job characteristics into two broad categories: job demands and job resources [20]. Job demands encompass the physical, psychological, social, or organizational aspects of work that require sustained effort and may lead to strain and exhaustion over time. In contrast, job resources refer to the physical, psychological, social, or organizational aspects that support goal achievement, reduce job demands, and stimulate personal growth and development. When job resources are sufficient, they can enhance motivation and engagement by fulfilling individuals’ psychological needs, thereby fostering positive work behaviors and outcomes [21]. Professional mission, as an important psychological resource, arises when individuals perceive their occupation as a source of purpose and meaning. Nurses with a strong sense of mission are more likely to demonstrate proactive attitudes, selfless dedication, and resilience in overcoming challenges, which helps them realize their professional value [14]. Work engagement, as a positive and persistent psychological state, serves as a buffer against the negative effects of job demands—such as stress, burnout, and compassion fatigue—while also promoting desirable work behaviors and performance [15]. Furthermore, to counteract the depletion caused by continuous job demands, mission-oriented nurses proactively mobilize psychological resources and adopt adaptive coping strategies, thereby maintaining and enhancing their performance levels. Previous studies have consistently shown that work engagement serves as a positive predictor of job performance, supporting behaviors and outcomes that align with organizational objectives [18]. Therefore, we propose the following research hypothesis:

H2: Job engagement mediates the relationship between nurses’ sense of professional mission and job performance.

### The moderating role of the spiritual climate

Spiritual climate is a positive workplace spiritual and cultural atmosphere felt by individuals, which is an available external supportive resource that can help individuals relieve work pressure and enhance organizational cohesion to better cope with work challenges [16]. An excellent spiritual climate can weaken burnout and negative emotions caused by high workloads in the long term, thus improving work efficiency and quality [22]. Previous studies have shown that the external environment positively impacts human motivation and positivity at work [17]. According to the Job Demands-Resources Model, job resources refer to material, psychological, social, or organizational factors that contribute to achieving job goals, alleviating job demands, and promoting personal growth and development [20]. The spiritual climate represents nurses’ shared perceptions of organizational values, respect, care, and support within the workplace. It constitutes a critical, organization-level social job resource that can predict enhanced job experiences, work well-being, and psychological empowerment [23]. An excellent mental climate can lead to more proactive participation of individuals at work and a better chance of realizing their professional values in the team. For example, Kazemipour et al. showed that mental climate positively affects nurses’ organizational citizenship behavior [24]. An excellent spiritual climate enhances individuals’ career development and sense of responsibility, enhancing dedication and organizational identity. Organizations with better spiritual climates also tend to have managers who are inclined to adopt inclusive leadership styles and humane management, with good communication channels and an atmosphere for the expression of ideas among organizational members, which can channel their physical strength and energy into activities that enhance the results of work behaviors. It has also been shown that a spiritual climate strengthens transformational leaders by increasing their awareness of the purpose and meaning of their work [25]. In a favorable mental climate, employees view work stressors as opportunities and challenges for career advancement rather than hindrances. As a result, a positive spiritual climate is more likely to activate their passion for their work and keep them energized and focused on their work to better achieve organizational goals. Prior studies have also shown that a positive hospital spiritual climate is significantly associated with high levels of nurses’ work engagement [26]. De et al. found that employees’ spiritual climate and work engagement moderated the effects of positive subjective behaviors on job performance, influencing their work behaviors and outcomes [27]. Based on this, we propose the following hypothesis:

H3: The spiritual climate can moderate the relationship between professional mission and work engagement.

**Fig 1:**
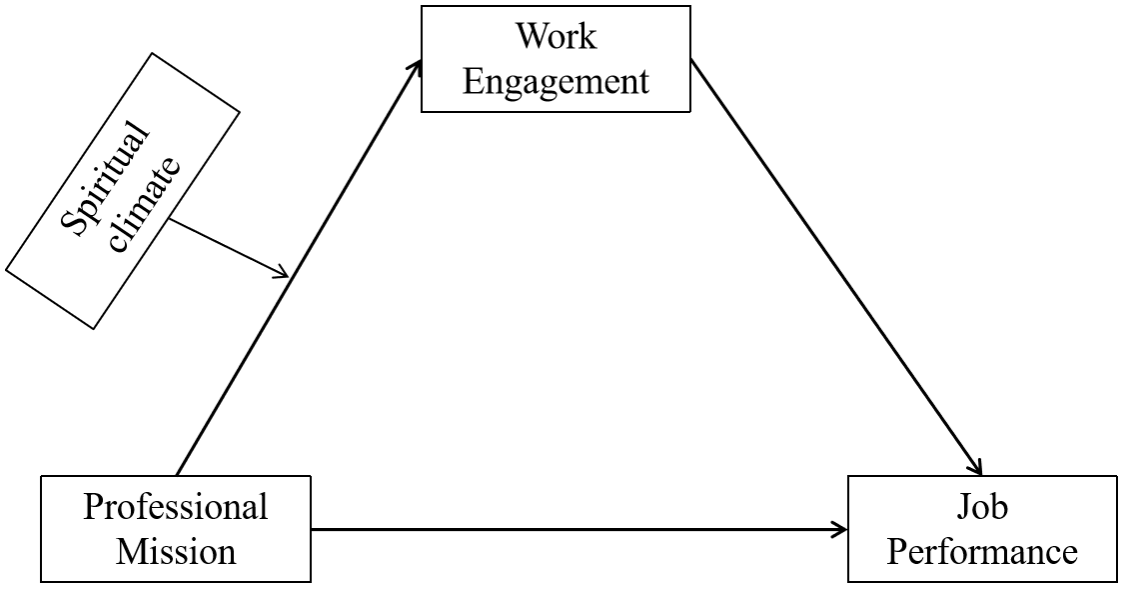
Analysis of the moderating role of spiritual climate in professional mission-work engagement-job performance.

In summary, the positive association between professional mission and job performance has been well-established. However, research examining the mediating role of work engagement and the moderating influence of spiritual climate in this relationship remains limited. As a key personal resource and form of intrinsic motivation, a strong professional sense of mission may enhance job performance by fostering work engagement among nurses. Meanwhile, spiritual climate, as a critical contextual factor, may moderate this mechanism by either strengthening or weakening the effect of professional sense of mission on work engagement. Grounded in self-determination theory and the job demands-resources model, this study constructs a moderated mediation model to investigate the mediating role of work engagement in the relationship between nurses’ professional sense of mission and job performance, as well as the moderating effect of spiritual climate. The findings aim to offer theoretical insights and practical implications for enhancing nurses’ job performance, improving the quality of nursing care, and promoting patient satisfaction. The proposed research framework is presented in Fig 1.

## Method

### Aims

The purpose of this study was to examine the role of clinical nurses’ sense of professional mission as a direct influence on job performance, as well as the mediating role of work engagement and the moderating role of spiritual climate.

### Design

This study was a cross-sectional survey.

### Participants

An online anonymous questionnaire survey of nurses from 15 tertiary hospitals and ten secondary hospitals in Sichuan Province, China, was conducted from February 2024 to March 2024 using a convenience sampling method.25 The hospitals surveyed were mainly from five regions of Sichuan Province (North Sichuan, South Sichuan, East Sichuan, South Sichuan, and Chengdu), with three tertiary hospitals and two secondary hospitals randomly selected from each region. All investigators signed an electronic informed consent form. Inclusion criteria: (1) clinical registered nurses; (2) more than one year in clinical nursing; (3) informed consent and voluntary participation in this study. Exclusion criteria: (1) internship, training, and further training nurses; (2) nurses who were not on duty, such as maternity leave, personal leave, and sick leave. Sample size was calculated using G*Power software (version 3.1) [28]. Based on Cohen’s standards [29], power was set at 0.90, alpha at 0.05, and effect size f²at 0.02. The study involved 21 independent variables. Using these parameters, G*Power calculated a required sample size of 528. Accounting for a 20% non-response rate, the calculated sample size was 660. The study ultimately collected 3287 questionnaires. After excluding 363 invalid responses (e.g., logical errors in demographic data, six or more consecutive identical answers, submission times under 2 minutes or over 30 minutes), 2924 valid questionnaires were retained.

### Measurements

#### Demographic information questionnaire.

The research team designed their own demographic information questionnaire based on the purpose of the study, which included a total of age, gender, marital status, and highest level of education.

#### Job Performance Scale.

The Job Performance Scale compiled by Borman et al. [30] and Chineseized and compiled by Wang et al. [31] was used. The scale includes two dimensions, task performance and contextual performance, with a total of 10 entries. The Likert 5-point scale was adopted, with scores ranging from 1 to 5 from “strongly disagree” to “strongly agree,” and the total score was 10–50, and the higher the score, the higher the job performance. This scale demonstrates good reliability and validity. Confirmatory factor analysis indicates a well-fitted single-factor structure, with a TLI of 0.900 and an RMSEA of 0.082. In this study, the Cronbach’s alpha coefficient for the scale was 0.947.

#### Professional Mission Scale.

The scale was compiled by Dobrow et al. [32] in 2011 and Zhang et al. [33] in 2015. The scale includes three dimensions of orientation and altruistic contribution, with a total of 10 entries. The scale is scored on a 5-point Likert scale, with each entry ranging from 1 to 5 points and the total score ranging from 11 to 55 points, with higher scores indicating a higher sense of professional mission. This scale demonstrates good reliability and validity. Confirmatory factor analysis indicates a well-fitted single-factor structure, with a TLI of 0.965, RMSEA of 0.038, and Cronbach’s alpha coefficient of 0.840. In this study, the Cronbach’s alpha coefficient for this scale was 0.968.

#### Work Engagement Scale.

The scale was compiled by Schaufeli et al. [34] in 2006 and Chineseized by Li et al. [35] in 2013. The scale consists of three dimensions: concentration, dedication, and vigor, with a total of 9 entries. The scale was scored on a 7-point Likert scale, with scores ranging from 0 to 6, from “never” to “every day,” and a total score of 0–54. The higher the score, the higher the level of work engagement. This scale demonstrates good reliability and validity. Confirmatory factor analysis indicates a well-fitted single-factor structure, with a Cronbach’s alpha coefficient of 0.930.

#### Spiritual Climate Scale.

The scale was developed by Doram et al. [36] and revised by Wu et al. [37] Chinese. The revision process adhered to Brislin’s translation-back-translation standard procedure. The scale is a one-dimensional scale with four items. The scale is a one-dimensional scale with four entries. It is rated on a 5-point Likert scale, with scores ranging from 1 to 5, from “strongly disagree” to “strongly agree,” and a total score of 4–20. The higher the score, the better the mental climate of the work environment is. The Chinese version of the spiritual climate scale demonstrates good reliability and validity, making it suitable for assessing Chinese nurses’ mental satisfaction with their work environment. This scale demonstrates good reliability and validity. Confirmatory factor analysis indicates a well-fitted single-factor structure, with TLI = 0.981 and RMSEA = 0.068. The scale exhibits a Cronbach’s alpha coefficient of 0.833, a split-half reliability coefficient of 0.800, and a test-retest reliability coefficient of 0.834. In this study, the Cronbach’s alpha coefficient for the scale was 0.955.

### Data collection

This study utilized electronically administered questionnaires designed and distributed through the WenJuanXing platform. Initially, the research team contacted the heads of nursing departments at the participating hospitals to secure informed consent and institutional authorization. A designated liaison officer was appointed at each hospital. All liaison officers then received unified training regarding the survey’s objectives, significance, procedures for completion, and important considerations. Following the training, the liaison officers distributed the questionnaire link within internal nursing work groups. Participants independently completed and submitted the surveys online. To ensure confidentiality, all responses were collected anonymously. Additionally, to maintain data integrity, all questionnaire items were set as mandatory, and each IP address was limited to a single submission. After the survey period ended, the collected responses were screened, and questionnaires exhibiting obvious response patterns or logical inconsistencies were excluded.

### Ethical considerations

The study followed the Declaration of Helsinki and was approved by the Ethics Committee of Deyang People’s Hospital (2024-04-016-K01). Prior to the formal commencement of the survey, the research team provided comprehensive details regarding the study’s objectives, methodology, and significance to the relevant institutional authorities and secured official approval from the hospital administration. The survey was administered under strict anonymity conditions. A standardized electronic informed consent form was presented on the initial page of the online questionnaire platform, which all participants were required to complete before proceeding. Participation was entirely voluntary, based on the principle of full autonomy. All respondents retained the right to withdraw from the study unconditionally at any point without facing any negative repercussions. No minors were included in this research. The investigators implemented stringent data confidentiality measures to ensure that all collected information remained anonymized and secure, thereby effectively protecting the privacy and personal data of all participants.

### Data analysis

Statistical analysis was performed using SPSS 26.0 software and PROCESS Macro. Frequency, mean, and standard deviation were used for descriptive analysis, and independent samples t-test with one-way ANOVA was used to explore the effect of general demographic information on nurses’ job performance. Pearson correlation analysis was used to analyze the relationship between professional mission, work engagement, spiritual climate, and job performance. Model 4 and Model 7 in the SPSS 26.0 macro program, were used to test the mediating role of work engagement and the moderating role of spiritual climate. The Bootstrap sample size was set at 5000, and the test level α = 0.05.

## Results

### Demographic characteristics of participants

Of the 2924 nurses, the average age was (31.93 ± 6.96) years. 95.5% of them were female, 70.2% were married nurses, 68.0% were nurses with bachelor’s degrees, and 63.8% were nurses from tertiary hospitals. The rest of the general information is shown in Table 1.

**Table 1:** Demographic characteristics of participants.

Variables	Mean (SD) or N (%)
Age	31.93 (6.96)
Gender	
Male	131 (4.48)
Female	2793 (95.52)
Marital and childbearing status	
Unmarried	787 (26.92)
Married but not having children	315 (10.77)
Married and having children	1737 (59.40)
Divorced or other	85 (2.91)
Highest level of education	
College and below	912 (31.19)
Undergraduate	1987 (67.95)
Master’s degree or above	25 (0.86)
Hospital level	
Tertiary	1059 (36.22)
Secondary	1865 (63.78)
Department	
Internal medicine system	739 (25.27)
Surgical system	667 (22.81)
Maternal and child system	414 (14.16)
Critical care system	172 (5.89)
Operating room systems	179 (6.12)
Outpatient and emergency system	310 (10.60)
Other	443 (15.15)
Professional title	
Nurse	642 (21.96)
Nurse practitioner	1231 (42.10)
Nurse supervisor	908 (31.05)
Deputy chief nurse and above	143 (4.89)
Position	
Clinical nurse	2658 (90.90)
Nursing team leader	236 (8.07)
Head nurse	30 (1.03)
Mode of employment	
Labor contract	2459 (84.10)
Professional preparation	465 (15.90)
Years of experience (year)	
≤ 3	546 (18.67)
4~<10	1051 (35.94)
10~<15	796 (27.22)
≥ 15	531 (18.16)
Average monthly personal income(RMB)	
<5000	1383 (47.30)
5000~<8000	1135 (38.82)
8000~<11000	317 (10.84)
≥ 11000	89 (3.04)
Whether on night shift	
Yes	1863 (63.71)
No	1061 (36.29)

### Comparison of job performance scores of clinical nurses with different general characteristics

Comparison of the job performance scores of clinical nurses with different ages, gender, marital and childbearing statuses, highest education level, hospital grade, department, title, employment mode, years of work experience, average monthly income of individuals, and whether they work shifts or not, the difference is statistically significant (*P* < 0.05); comparison of the job performance scores of clinical nurses with different positions, the difference is not statistically significant (*P* > 0.05). See Table 2.

**Table 2:** Comparison of job performance scores of clinical nurses with different general characteristics.

Variables	Job performance(M ± SD)	*t/F*	*P*
Age		5.446	<0.001
Gender		−2.103	0.036
Male	42.02 ± 6.90		
Female	43.20 ± 6.20		
Marital and childbearing status		31.621	<0.001
Unmarried	41.60 ± 6.51		
Married but not having children	42.05 ± 6.45		
Married and having children	43.99 ± 5.93		
Divorced or other	44.22 ± 5.90		
Highest level of education		13.425	<0.001
College and below	42.29 ± 6.53		
Undergraduate	43.55 ± 6.08		
Master’s degree or above	41.76 ± 4.88		
Hospital level		−3.454	0.001
Tertiary	42.61 ± 6.36		
Secondary	43.44 ± 6.15		
Department		7.372	<0.001
Internal medicine system	42.85 ± 6.24		
Surgical system	43.13 ± 6.56		
Maternal and child system	43.81 ± 6.08		
Critical care system	40.62 ± 6.55		
Operating room systems	42.89 ± 5.96		
Outpatient and emergency system	44.15 ± 6.07		
Other	43.41 ± 5.70		
Professional title		40.885	<0.001
Nurse	41.03 ± 6.80		
Nurse practitioner	43.16 ± 6.13		
Nurse supervisor	44.40 ± 5.74		
Deputy chief nurse and above	44.55 ± 5.01		
Position		2.798	0.061
Clinical nurse	43.08 ± 6.32		
Nursing team leader	43.58 ± 5.27		
Head nurse	45.47 ± 5.66		
Mode of employment		−2.366	0.018
Labor contract	43.02 ± 6.26		
Professional preparation	43.77 ± 6.11		
Years of experience (year)		53.097	<0.001
≤ 3	40.46 ± 6.86		
4~<10	42.43 ± 6.29		
10~<15	44.27 ± 5.69		
≥ 15	45.04 ± 5.33		
Average monthly personal income(RMB)		13.457	<0.001
< 5000	42.49 ± 6.46		
5000~<8000	43.43 ± 6.26		
8000~<11000	44.46 ± 5.20		
≥ 11000	45.02 ± 4.22		
Whether on night shift		−6.630	<0.001
Yes	42.57 ± 6.43		
No	44.15 ± 5.75		

### Scores and correlation analysis of clinical nurses’ job performance, professional mission, work engagement, and spiritual climate

In this study, the mean score of clinical nurses’ job performance entries was (4.31 ± 0.62), the mean score of professional mission entries was (3.96 ± 0.76), the mean score of work engagement entries was (3.74 ± 0.79), and the mean score of spiritual climate entries was (4.18 ± 0.66). The results of Pearson correlation analysis showed that the mean scores of job performance and professional mission (r = 0.604, *P* < 0.01), work engagement (r = 0.576, *P* < 0.01), and spiritual climate (r = 0.552, *P* < 0.01) were positively correlated; professional mission was positively correlated with work engagement (r = 0.863, *P* < 0.01), and spiritual climate (r = 0.711, *P* < 0.01); work engagement was positively correlated with spiritual climate (r = 0.767, *P* < 0.01) were positively correlated. According to Cohen’s criteria [38], this effect size qualifies as a large effect. See Table 3.

**Table 3:** Descriptive statistics and correlations among variables.

Variables	Entry mean score (M ± SD)	1	2	3	4
1. Job performance	4.31 ± 0.62	1			
2. Professional mission	3.96 ± 0.76	0.604**	1		
3. Work engagement	3.74 ± 0.79	0.576**	0.863**	1	
4. Spiritual climate	4.18 ± 0.66	0.552**	0.711**	0.767**	1

**P < 0.01.

### Analysis of the mediating effect of clinical nurses’ work engagement between professional mission and job performance

**Fig 2:**
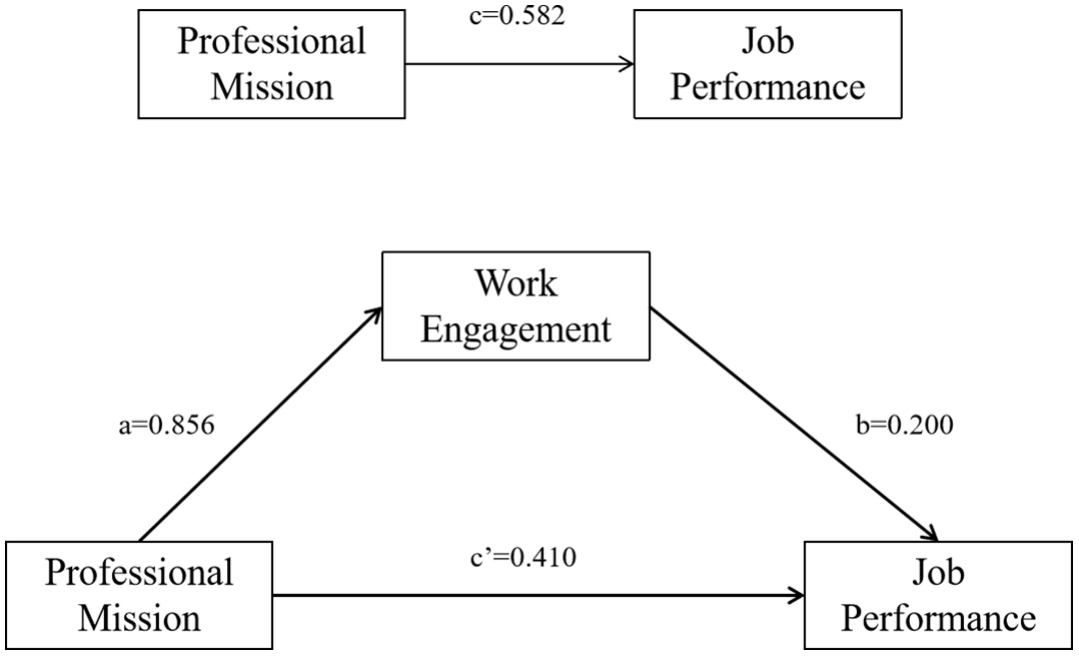
The mediating effect of work engagement between professional mission and job performance.

The results of the multiple linear regression analysis of socio-demographic statistics showed that age, mode of employment, and years of work experience had a significant effect on job performance. Therefore, age, mode of employment, and years of work experience were used as control variables. All variables were standardized, and mediated effects analysis was conducted using Model 4 in the PROCESS 4.1 macro program. The results showed that after controlling for the effects of age, mode of employment, and years of work experience, clinical nurses’ sense of professional mission positively predicted job performance (β = 0.410, SE = 0.029, t = 14.270, *P* < 0.001) and positively predicted work engagement (β = 0.856, SE = 0.10, t = 89.418, *P* < 0.001); work engagement positively predicts clinical nurse job performance (β = 0.200, SE = 0.029, t = 6.973, *P* < 0.001). Further, using the bias-corrected Bootstrap method with 5,000 repetitive samples, the results showed that the 95% confidence intervals for the direct effect of clinical nurses’ sense of professional mission on job performance and the mediating effect of work engagement did not contain 0. The direct effect of a sense of professional mission and the mediating effect of work engagement accounted for 70.4% and 29.6% of the total effect, respectively. See Table 4 and Fig 2.

**Table 4:** Mediating effects of work engagement between professional mission and job performance.

Effects	β	SE	*t*	*P*	95%CI	Relative effect ratio
Total effect	0.582	0.015	38.820	<0.001	(0.552, 0.611)	–
Direct effect	0.410	0.029	14.270	<0.001	(0.354, 0.466)	70.4%
Indirect effect	0.172	0.027	–	–	(0.118, 0.225)	29.6%

### Analysis of the moderating role of clinical nurses’ spiritual climate in professional mission-work engagement-job performance

**Fig 3:**
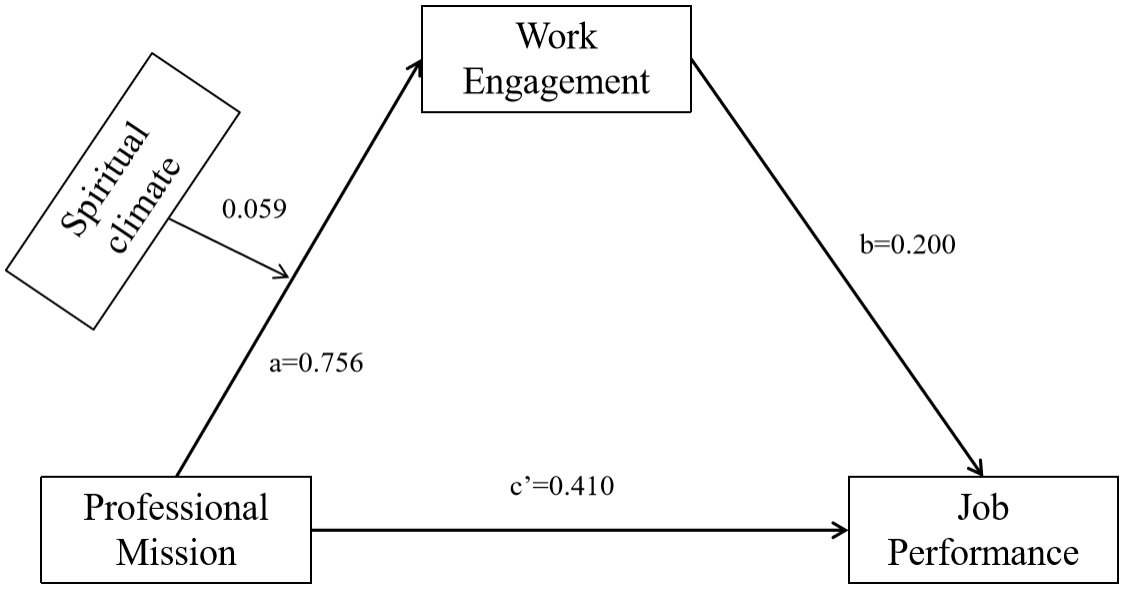
Analysis of the moderating role of spiritual climate in professional mission-work engagement-job performance.

Based on the research hypotheses of this study, the mediated effects model with moderation was tested using Model 7 in the PROCESS 4.1 macro program. The results showed that after controlling for the effects of age, employment mode, and years of experience, sense of professional mission positively predicted job performance (β = 0.410, SE = 0.029, t = 14.270, *P* < 0.001), positively predicted job engagement (β = 0.756, SE = 0.013, t = 57.129, *P* < 0.001), and professional mission and spiritual climate interaction term positively predicted work engagement (β = 0.059, SE = 0.008, t = 6.997, *P* < 0.001); and work engagement positively predicted clinical nurses’ job performance (β = 0.200, SE = 0.029, t = 6.973, *P* < 0.001). Thus, spiritual climate, as a moderating variable, enhanced the positive predictive effect of professional mission on work engagement. See Table 5 and Fig 3.

**Table 5:** Analysis of mediating moderating effects of work engagement and spiritual climate between clinical nurses’ sense of professional mission and job performance.

Dependent Variables	Independent Variables	*R* ^2^	F	β	SE	*t*	*P*	95%CI
Job performance	Age	0.379	445.712**	0.001	0.004	0.313	0.754	−0.007 ~ 0.009
	Mode of employment			−0.117	0.046	−2.546	0.011	−0.207 ~ −0.027
	Years of experience			0.124	0.027	4.542	<0.001	0.070 ~ 0.177
	Professional mission			0.582	0.015	38.820	<0.001	0.552 ~ 0.611
Work engagement	Age	0.758	1525.652**	0.006	0.003	2.445	0.015	0.001 ~ 0.011
	Mode of employment			−0.043	0.029	−1.482	0.138	−0.099 ~ 0.014
	Years of experience			−0.006	0.017	−0.335	0.737	−0.039 ~ 0.028
	Professional mission			0.756	0.013	57.129	<0.001	−0.280 ~ −0.081
	Spiritual climate			0.143	0.013	10.853	<0.001	0.117 ~ 0.169
	Professional mission*Spiritual climate			0.059	0.008	6.997	<0.001	0.043 ~ 0.076
Job performance	Age	0.389	372.112**	0.000	0.004	0.014	0.989	−0.008 ~ 0.008
	Mode of employment			−0.107	0.046	−2.344	0.019	−0.196 ~ −0.017
	Years of experience			0.125	0.027	4.621	<0.001	0.072 ~ 0.178
	Professional mission			0.410	0.029	14.270	<0.001	0.354 ~ 0.466
	Work engagement			0.200	0.029	6.973	<0.001	0.144 ~ 0.257

The mediated effect value of work engagement was 0.140 with 95% CI [0.096, 0.183] when the spiritual climate was at a low level, 0.151 with 95% CI [0.105, 0.197] when the spiritual climate was at an intermediate level (M), and 0.163 when the mental climate was at a high level, with a 95% CI [0.113, 0.211]. It indicates that spiritual climate moderates the mediating role of the work head chart between a sense of professional mission and job performance, which is manifested in the fact that the higher the level of spiritual climate of clinical nurses, the stronger the indirect effect of a sense of professional mission affecting job performance through work engagement, and vice versa, the weaker this indirect effect. See Table 6.

**Table 6:** Mediating effects of work engagement at different levels of spiritual climate.

	Spiritual climate	Effect size	Boot SE	Boot 95% CI LLCI	Boot 95% CI ULCI
Moderated mediating effects	(M-1SD)	0.140	0.022	0.096	0.183
	(M)	0.151	0.023	0.105	0.197
	(M ＋ 1SD)	0.163	0.025	0.113	0.211
Index of moderated mediation		0.012	0.003	0.007	0.017

### Results of simple slope analysis

**Fig 4:**
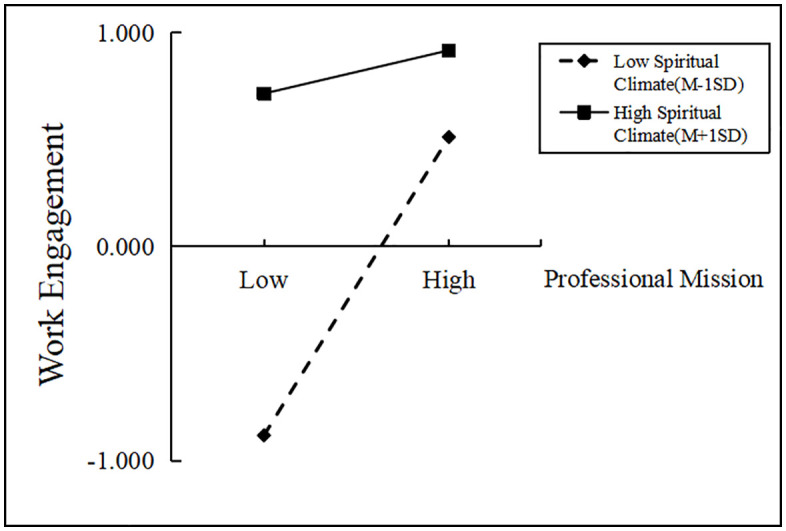
Simple slopes of professional mission on work engagement at different levels of spiritual climate.

Further results of simple slope analysis showed that when the mental climate was at a low level (M-1SD), the positive predictive effect of clinical nurses’ sense of professional mission on work engagement was significant (β = 0.696, SE = 0.017, t = 42.153, *P* < 0.001), and when the spiritual climate was at a high level (M + 1SD), the positive predictive prediction was also significant (β = 0.815, SE = 0.015, t = 54.818, *P* < 0.001). This indicates that the predictive effect of a sense of professional mission on work engagement is gradually increasing as the level of spiritual climate increases. See Fig 4.

## Discussion

This study constructed a mediating effect model of the relationship between the sense of professional mission and nurses’ job performance and explored the relationship between the sense of professional mission and job performance, as well as the mediating role of work engagement and the moderating role of spiritual climate. On the one hand, it is shown that a sense of professional mission affects nurses’ job performance through the mediating role of work engagement. On the other hand, it is shown that spiritual climate plays a moderating role in the first half of the pathway of the mediation model of nurses’ sense of professional mission, affecting job performance through work engagement. A high level of spiritual climate enhances the effect of a sense of professional mission on work engagement and thus enhances job performance. This study provides a theoretical basis for targeted intervention strategies to further enhance nurses’ job performance in the context of the severe global shortage of nurses and the increasing demand for quality of healthcare services.

In this study, the professional mission score was consistent with the study by Li et al. [39] and higher than that of Jin [40] and Zhang et al. [41]. This difference may be related to the individual factors of the survey respondents, such as education, job title, and income level. Nurses with higher academic qualifications have better career development prospects, have more advantages in title and job promotion, and also reap decent pay performance. As a result, they are more motivated to perceive the purpose and meaning of their work to achieve organizationally relevant behaviors and outcomes. Clinical nurses’ work engagement is at a medium level, which is consistent with the findings of Dong et al. [42]. It indicates that the level of Chinese nurses’ work engagement needs to be further improved, which may be related to the fact that the shortage of nursing human resources is particularly prominent in China, where nurses are required to undertake clinical, teaching, and scientific research work at the same time, and most of them are required to work night shifts, which results in nurses being in a long-term high-load and high-stress work environment and are susceptible to high burnout and turnover intention, thus affecting their work vitality, focus, and dedication. In addition, the mental climate scores were higher than the results of Cruz et al. [26], which is consistent with the findings of Wang et al. [23] in China. It may be related to the differences in economic, cultural, and healthcare work environments in different countries. Finally, nurses’ job performance in this study was at a high level, consistent with the results of Song et al [43]. This indicates that the overall high level of nurse job performance in China may be related to the rapid development of nursing education, which has significantly improved nurses’ professional skills and abilities. In addition, the Chinese governmental departments have conducted a series of policy explorations to improve the job performance of medical personnel, such as pay performance reform, title promotion reform, work environment improvement, and healthcare service concept change, which have stimulated nurses’ work enthusiasm and motivation and influenced their job performance.

The results of this study showed that a sense of professional mission had a significant positive predictive effect on job performance, confirming research hypothesis 1 and consistent with the results of previous studies [19,44]. In other words, nurses with a strong sense of professional mission are better able to closely link their personal goals with organizational goals, actively seek solutions to problems at work, and overcome unattainable organizational goal outcomes, thus improving job performance. According to self-determination theory, individuals tend to make behavioral choices based on evaluating their own value needs and external environmental information. This process emphasizes the potential influence of intrinsic motivation on individual behavior and outcomes. For nurses, perceiving the achievement of organizational goals and tasks as an expression of personal value and meaning may be linked to the formation of their intrinsic motivation [13]. When nurses possess a high level of professional mission, work objectives are often imbued with deeper personal value and social contribution significance. This may further stimulate positive attitudes and creative performance in their work, correlating with improved job performance. Moreover, a heightened professional mission may also mitigate burnout, reduce turnover intentions, and strengthen psychological resilience among nurses. The combined effect of these factors can positively influence work efficiency and service quality [39,45]. Therefore, nursing managers should focus on fostering nurses’ intrinsic motivation and enhancing their professional identity and sense of mission in management practices. This can be achieved by cultivating a supportive organizational atmosphere and providing development platforms and opportunities that facilitate professional growth. Such efforts will further promote nurses’ sense of accomplishment and satisfaction at work, thereby positively correlating with increased work engagement.

The results of this study found that work engagement mediates the relationship between nurses’ sense of professional mission and job performance, with the mediating effect accounting for 29.6% of the total effect, confirming research hypothesis 2. Specifically, when nurses possess a strong sense of professional mission, it can stimulate their enthusiasm for their work, thus showing positive psychological qualities such as vigor, concentration, and dedication at work and generating a high level of work engagement. Compared with nurses with a low level of work engagement, nurses with a higher level of work engagement tend to be positive enough to face challenges and pressures at work, reduce the impact of negative emotions such as emotional exhaustion and anxiety on physical and mental health, and thus enhance work well-being and satisfaction, and improve work performance [46]. Cui, et al.’s study, also shows that a sense of professional mission has a strong facilitating effect on work engagement and job performance. In the practice of organizational management, it is conducive to the enhancement of job performance to play the dynamic role of employees’ sense of professional mission. Similar to Fashafsheh et al. [47], who found emotional intelligence enhances performance, our study highlights intrinsic motivation (professional mission) as a key driver of engagement. Studies by Ayed et al. also indicate that the work environment is closely related to nurses’ job satisfaction [48]. Meanwhile, based on self-determination theory, when nurses feel the value and meaning brought by nursing work, they will be more determined in their career choices and hope to gain a greater sense of accomplishment in their work. Previous studies have shown that work engagement has a positive impact on nurses’ work behavior, work outcomes, work well-being, and organizational commitment [49–51]. Therefore, nursing managers should pay attention to nurses’ career development planning, strengthen nursing professional connotation and significance training, and enhance nurses’ sense of professional identity and value; in addition, sunny and decent salary returns, reasonable human resource allocation, and safe and comfortable working environment are conducive to enhancing nurses’ motivation and dedication to their work, and thus improving their job performance.

This study further demonstrates that the pathway through which professional sense of mission influences nursing performance via work engagement is moderated by spiritual climate, thereby supporting research hypothesis 3. Specifically, spiritual climate significantly strengthens the positive relationship between professional mission and work engagement—that is, the predictive effect of professional mission on work engagement becomes more pronounced under more favorable spiritual climate conditions. According to the job demands-resources model [20], workplace morale serves as a critical organizational resource that offers employees psychological support and enhances their sense of meaning at work. In a positive spiritual climate, individuals are more likely to express their opinions freely, which strengthens their sense of involvement and ownership. This reinforces their perception of self-worth and the significance of their work, mitigates negative emotions associated with work-related stress, and ultimately promotes higher work engagement and improved performance. Moreover, a supportive workplace atmosphere enhances organizational cohesion and increases perceived organizational support, enabling nurses to obtain more extensive social resources. This helps them cope more proactively with challenges and setbacks in the work environment [22]. Although notable differences exist between Chinese and Western cultural contexts, fundamental psychological needs—such as the desire for meaningful work, respect, and support—may exhibit cross-cultural commonality. However, the manifestation and fulfillment of these needs can vary across cultures. China’s unique Confucian cultural tradition plays a pivotal role in shaping nurses’ perceptions of the psychological climate. Against the backdrop of Western individualism, “respect”may emphasize the recognition of individual autonomy and uniqueness. In contrast, within China’s collectivist context, grounded in Confucian thought, the psychological climate’s dimensions of “respect,” “support,” and “harmony” bear the profound imprint of Confucian principles [22]. Therefore, nursing administrators should fully recognize the critical role of psychological climate in the mechanism by which professional mission influences work performance through job engagement. This can be achieved by implementing systematic “spiritual leadership” training modules and establishing a “humanistic care mentorship program” to actively foster a supportive and inclusive workplace culture. For instance, pairing newcomers and nurses at high risk of burnout with experienced mentors can provide regular emotional support, career guidance, and cultural transmission, helping them develop a sense of belonging within the collective. Second, establishing peer support programs, advocating for humanistic care, and encouraging team mutual aid and meaning perception can enhance organizational recognition and support for nurses’ intrinsic motivation. This approach fully unleashes nurses’ initiative and creativity, effectively boosting their work vitality, focus, and dedication, thereby enhancing their performance further.

## Limitations

Although this study confirmed the relationships among professional mission, work engagement, spiritual climate, and job performance, several limitations should be acknowledged. First, this study employed convenience sampling, with all participants originating from Sichuan Province, China, which to some extent limits the external validity of the findings. Although Sichuan Province is highly representative of vast regions across China, particularly the central and western areas, its unique regional culture, level of economic development, and distribution of healthcare resources imply that further validation is needed to determine whether the model constructed in this study remains valid in economically developed eastern regions or northern cultural contexts. Future research should conduct multi-regional, multi-center, large-sample studies. Samples should be systematically drawn from different geographical and economic zones across eastern, northern, and southern China to validate the model’s nationwide applicability. More importantly, multi-group comparative analyses should investigate whether macro-level variables, such as regional culture and economic development levels, play moderating roles within the model. Second, the cross-sectional design of this study captures variable relationships only at a specific point in time, which prevents the establishment of causal inferences. Longitudinal studies tracking variables over time are recommended to examine causal pathways and dynamic trends. Moreover, although online surveys were utilized for data collection, this method may be susceptible to subjective interpretation by respondents, potentially affecting the accuracy of the measured constructs. Future studies would benefit from incorporating objective indicators and triangulating online measures with in-person interviews to enhance the authenticity and comprehensiveness of the data. Finally, nurse job performance is a multifactorial outcome influenced by a range of individual factors (e.g., education level, professional title, personality, health status) and contextual elements (e.g., leadership style, workload). Therefore, subsequent research should further develop the theoretical model and investigate the key determinants of job performance from an integrated, multi-dimensional perspective. Finally, this study employed self-report questionnaires for data collection, which carries the risk of common method bias. Although we implemented procedural safeguards and conducted Harman’s single-factor test post-data collection, revealing that the first non-rotated factor did not account for substantial variance, this does not entirely rule out the potential influence of common method bias. Given the limited sensitivity of Harman’s test, future research may employ marker variables or utilize longitudinal designs to more effectively address this issue.

## Conclusion

The results of this study indicate that clinical nurses’ job performance is at a high level, that sense of professional mission is a significant positive predictor of nurses’ job performance, and that work engagement mediates the relationship between nurses’ sense of professional mission and job performance, and that spiritual climate moderates the relationship between sense of professional mission and work engagement. The model elucidated how nurses’ sense of professional mission affects job performance (mediating role of work engagement). It also identifies the conditions under which a sense of professional mission has a significant positive predictive effect on work engagement (moderating role of spiritual climate). It can help nursing managers recognize the importance of nurses’ work value orientation and positive workplace spiritual climate in improving nurses’ motivation and creativity to better assist nurses in improving their job performance.

## Supporting information

S1 File Raw data. (XLSX)

## References

[1] Marć M, Bartosiewicz A, Burzyńska J, Chmiel Z, Januszewicz P. A nursing shortage - a prospect of global and local policies. Int Nurs Rev. 2019;66(1):9–16. doi: 10.1111/inr.12473 30039849

[2] International Council of Nurses. Recover to rebuild: investing in the nursing workforce for health system effectiveness, a report prepared by Buchan, J. and Catton H. 2023. Available from: https://www.icn.ch/system/files/2023–03/ICN_Recover-to-Rebuild_report_EN

[3] World Health Organization. State of the world’s nursing 2020. 2023. Available from: https://www.who.int/publications/i/item/9789240003279

[4] Wan Q, Li Z, Zhou W, Shang S. Effects of work environment and job characteristics on the turnover intention of experienced nurses: The mediating role of work engagement. J Adv Nurs. 2018;74(6):1332–41. doi: 10.1111/jan.13528 29350781

[5] Aung Po WW, Wichaikhum OA, Abhicharttibutra K, Suthakorn W. Factors predicting job performance of nurses: A descriptive predictive study. Int Nurs Rev. 2023. 10.1111/inr.12873 37571966

[6] Zhang N, Xu D, Li J, Xu Z. Effects of role overload, work engagement and perceived organisational support on nurses’ job performance during the COVID-19 pandemic. J Nurs Manag. 2022;30(4):901–12. doi: 10.1111/jonm.13598 35293044 PMC9115180

[7] Abbasi M, Monazzam MR, Karanika-Murray M, Shamsipour M, Arabalibeik H. Development and validation of an individual job performance questionnaire (IJPQ). Work. 2022;73(1):309–20. doi: 10.3233/WOR-211004 35871384

[8] Dyrbye LN, Shanafelt TD, Johnson PO, Johnson LA, Satele D, West CP. A cross-sectional study exploring the relationship between burnout, absenteeism, and job performance among American nurses. BMC Nurs. 2019;18:57. doi: 10.1186/s12912-019-0382-7 31768129 PMC6873742

[9] Wu C, Fu M-M, Cheng S-Z, Lin Y-W, Yan J-R, Wu J, et al. Career identity and career success among Chinese male nurses: The mediating role of work engagement. J Nurs Manag. 2022;30(7):3350–9. doi: 10.1111/jonm.13782 36056581 PMC10087454

[10] Kohnen D, De Witte H, Schaufeli WB, Dello S, Bruyneel L, Sermeus W. What makes nurses flourish at work? How the perceived clinical work environment relates to nurse motivation and well-being: A cross-sectional study. Int J Nurs Stud. 2023;148:104567. doi: 10.1016/j.ijnurstu.2023.104567 37837704

[11] Labrague LJ, Al Sabei SD, AbuAlRub RF, Burney IA, Al Rawajfah O. Authentic leadership, nurse-assessed adverse patient events and quality of care: The mediating role of nurses’ safety actions. J Nurs Manag. 2021;29(7):2152–62. doi: 10.1111/jonm.13356 33960043

[12] Fiorini LA, Houdmont J, Griffiths A. Nurses’ perceived work performance and health during presenteeism: Cross-sectional associations with personal and organisational factors. J Nurs Manag. 2022;30(5):O37–45. doi: 10.1111/jonm.13065 32506664

[13] Flannery M. Self-Determination Theory: Intrinsic Motivation and Behavioral Change. Oncol Nurs Forum. 2017;44(2):155–6. 28222078 10.1188/17.ONF.155-156

[14] Cao Y, Liu J, Liu K, Yang M, Liu Y. The mediating role of organizational commitment between calling and work engagement of nurses: A cross-sectional study. Int J Nurs Sci. 2019;6(3):309–14. doi: 10.1016/j.ijnss.2019.05.004 31508452 PMC6722477

[15] Kulikowski K. One, two or three dimensions of work engagement? Testing the factorial validity of the Utrecht Work Engagement Scale on a sample of Polish employees. Int J Occup Saf Ergon. 2019;25(2):241–9. doi: 10.1080/10803548.2017.1371958 28849984

[16] Cruz JP, Alquwez N, Mesde JH, Almoghairi AMA, Altukhays AI, Colet PC. Spiritual climate in hospitals influences nurses’ professional quality of life. J Nurs Manag. 2020;28(7):1589–97. doi: 10.1111/jonm.13113 32743944

[17] Tong L. Relationship between meaningful work and job performance in nurses. Int J Nurs Pract. 2018;24(2):e12620. doi: 10.1111/ijn.12620 29368384

[18] Ziedelis A. Perceived Calling and Work Engagement Among Nurses. West J Nurs Res. 2019;41(6):816–33. doi: 10.1177/0193945918767631 29591587

[19] Zhou X, Zhang Y, Wang Y, Wang H, Sun S, Huang X. The impact of medical staff’s character strengths on job performance in Hangzhou hospitals. Front Psychol. 2023;14:1291851. doi: 10.3389/fpsyg.2023.1291851 38078217 PMC10701392

[20] Bakker AB, Demerouti E. Job demands-resources theory: Taking stock and looking forward. J Occup Health Psychol. 2017;22(3):273–85. doi: 10.1037/ocp0000056 27732008

[21] Bakker AB, Demerouti E. Job demands-resources theory: Frequently asked questions. J Occup Health Psychol. 2024;29(3):188–200. doi: 10.1037/ocp0000376 38913705

[22] Zhang Y, Wu X, Wan X, Hayter M, Wu J, Li S, et al. Relationship between burnout and intention to leave amongst clinical nurses: The role of spiritual climate. J Nurs Manag. 2019;27(6):1285–93. doi: 10.1111/jonm.12810 31144776

[23] Wang X, Xia Y, Gou L, Wen X. Exploring the influence of the spiritual climate on psychological empowerment among nurses in China: a cross-sectional study. BMC Nurs. 2024;23(1):374. doi: 10.1186/s12912-024-02011-x 38831307 PMC11145847

[24] Kazemipour F, Mohamad Amin S, Pourseidi B. Relationship between workplace spirituality and organizational citizenship behavior among nurses through mediation of affective organizational commitment. J Nurs Scholarsh. 2012;44(3):302–10. doi: 10.1111/j.1547-5069.2012.01456.x 22804973

[25] Wu X, Hayter M, Lee AJ, Yuan Y, Li S, Bi Y, et al. Positive spiritual climate supports transformational leadership as means to reduce nursing burnout and intent to leave. J Nurs Manag. 2020;28(4):804–13. doi: 10.1111/jonm.12994 32145113

[26] Cruz JP, Alquwez N, Balay-Odao E. Work engagement of nurses and the influence of spiritual climate of hospitals: A cross-sectional study. J Nurs Manag. 2022;30(1):279–87. doi: 10.1111/jonm.13492 34619805

[27] De Carlo A, Dal Corso L, Carluccio F, Colledani D, Falco A. Positive Supervisor Behaviors and Employee Performance: The Serial Mediation of Workplace Spirituality and Work Engagement. Front Psychol. 2020;11:1834. doi: 10.3389/fpsyg.2020.01834 32793085 PMC7393218

[28] Faul F, Erdfelder E, Lang A-G, Buchner A. G*Power 3: a flexible statistical power analysis program for the social, behavioral, and biomedical sciences. Behav Res Methods. 2007;39(2):175–91. doi: 10.3758/bf03193146 17695343

[29] Cohen J. Chapter 4 - Differences between Correlation Coefficients. In: Cohen J, editor. Statistical Power Analysis for the Behavioral Sciences. New York: Academic Press; 1977. p. 109–143.

[30] Borman WC, Motowidlo SJ. Expanding the Criterion Domain to Include Elements of Contextual Performance. In: Schmitt N, Borman WC, editors. Personnel Selection in Organization. San Francisco: Jossey-Bass; 1993.

[31] Wang H, Li XX, Luo SQ. Validation of a two-factor performance model of task performance and situational performance. Chinese J Manag Sci. 2003;(04):80–5.

[32] Dobrow SR, Tosti‐Kharas J. Calling: the development of a scale measure. Personnel Psychol. 2011;64(4):1001–49. doi: 10.1111/j.1744-6570.2011.01234.x

[33] Zhang CY. Occupational sense of purpose: structure, measurement, and its link to well-being [PhD]. 2015.

[34] Schaufeli WB, Bakker AB, Salanova M. The measurement of work engagement with a short questionnaire. Educ Psychol Meas. 2013;66(4):701–16.

[35] Li FY, Zhao JL, Zhang P, Liu JW, Lian YL, Wang TT. Evaluation of the Reliability and Validity of the Chinese Version of the Public Security Police Work Engagement Scale. China Public Health. 2013;29(01):97–9.

[36] Doram K, Chadwick W, Bokovoy J, Profit J, Sexton JD, Sexton JB. Got spirit? The spiritual climate scale, psychometric properties, benchmarking data and future directions. BMC Health Serv Res. 2017;17(1):132. doi: 10.1186/s12913-017-2050-5 28189142 PMC5303307

[37] Wu XX, Zhang Y, Wu JF, Wang XJ, Hu Y, Liu YB, et al. A reliability study of the Chinese version of the Mental Climate Short-form Scale. Nursing Research. 2019;33(14):2396–9.

[38] Cohen J. A power primer. Psychol Bull. 1992;112(1):155–9. doi: 10.1037//0033-2909.112.1.155 19565683

[39] Li C, Cui X, Zhao Y, Xin Y, Pan W, Zhu Y. Missed Nursing Care as a Mediator in the Relationship between Career Calling and Turnover Intention. Int Nurs Rev. 2024;71(1):62–8. doi: 10.1111/inr.12842 37079658

[40] Jin T, Zhou Y, Zhang L. Job stressors and burnout among clinical nurses: a moderated mediation model of need for recovery and career calling. BMC Nurs. 2023;22(1):388. doi: 10.1186/s12912-023-01524-1 37853383 PMC10583433

[41] Zhang Y, Kuang D, Zhang B, Liu Y, Ren J, Chen L, et al. Association between hopelessness and job burnout among Chinese nurses during the COVID-19 epidemic: The mediating role of career calling and the moderating role of social isolation. Heliyon. 2023;9(6):e16898. doi: 10.1016/j.heliyon.2023.e16898 37303510 PMC10245282

[42] Dong X, Lu H, Wang L, Zhang Y, Chen J, Li B, et al. The effects of job characteristics, organizational justice and work engagement on nursing care quality in China: A mediated effects analysis. J Nurs Manag. 2020;28(3):559–66. doi: 10.1111/jonm.12957 31954085

[43] Song J, Shi X, Zheng X, Lu G, Chen C. The impact of perceived organizational justice on young nurses’ job performance: a chain mediating role of organizational climate and job embeddedness. BMC Nurs. 2024;23(1):231. doi: 10.1186/s12912-024-01898-w 38584272 PMC10999088

[44] Wu G, Hu Z, Zheng J. Role stress, job burnout, and job performance in construction project managers: The moderating role of career calling. Int J Environ Res Public Health. 2019;16(13). 10.3390/ijerph16132394 PMC6651169 31284496

[45] Xie SJ, Jing J, Li R, Yan SQ, Yu G, Xu ZJ. The impact of career calling on nurse burnout: A moderated mediation model. Int Nurs Rev. 2024. 10.1111/inr.12957 38477788

[46] Topa G, Aranda-Carmena M. Job Crafting in Nursing: Mediation between Work Engagement and Job Performance in a Multisample Study. Int J Environ Res Public Health. 2022;19(19):12711. doi: 10.3390/ijerph191912711 36232011 PMC9566469

[47] Fashafsheh IH, Eqtait FA, Hammad BM, Ayed AJ, Salameh BS. The impact of emotional intelligence on work performance among ICU nurses in Palestine: a cross-sectional study. BMC Nurs. 2025;24(1):413. doi: 10.1186/s12912-025-03068-y 40221734 PMC11993981

[48] Ayed A, Abu Ejheisheh M, Aqtam I, Batran A, Farajallah M. The Relationship Between Professional Quality of Life and Work Environment Among Nurses in Intensive Care Units. Inquiry. 2024;61:469580241297974. doi: 10.1177/00469580241297974 39520216 PMC11550503

[49] De Los Santos JAA, Labrague LJ. Job engagement and satisfaction are associated with nurse caring behaviours: A cross-sectional study. J Nurs Manag. 2021;29(7):2234–42. doi: 10.1111/jonm.13384 34021940

[50] Han S. Nurses’ job crafting, work engagement, and well-being: a path analysis. BMC Nurs. 2023;22(1):405. doi: 10.1186/s12912-023-01573-6 37904210 PMC10614409

[51] Al-Dossary RN. Leadership Style, Work Engagement and Organizational Commitment Among Nurses in Saudi Arabian Hospitals. J Healthc Leadersh. 2022;14:71–81. doi: 10.2147/JHL.S365526 35698661 PMC9188332

